# Dynamic Response of CoSb_2_O_6_ Trirutile-Type Oxides in a CO_2_ Atmosphere at Low-Temperatures

**DOI:** 10.3390/s140915802

**Published:** 2014-08-26

**Authors:** Alex Guillén-Bonilla, Verónica-María Rodríguez-Betancourtt, Martín Flores-Martínez, Oscar Blanco-Alonso, Juan Reyes-Gómez, Lorenzo Gildo-Ortiz, Héctor Guillén-Bonilla

**Affiliations:** 1 Materials Science Graduate School, CUCEI (Centro Universitario de Ciencias Exactas e Ingenierías), University of Guadalajara, Blvd. M. García Barragán 1421, Guadalajara, Jalisco 44410, Mexico; E-Mails: veronica.rodriguez@red.cucei.udg.mx (V.-M.R.-B.); doctorado.materiales@cucei.udg.mx (M.F.-M.); 2 Physics Department, CUCEI (Centro Universitario de Ciencias Exactas e Ingenierías), University of Guadalajara, Blvd. M. García Barragán 1421, Guadalajara, Jalisco 44410, Mexico; E-Mail: oscar.blanco@cucei.udg.mx; 3 Faculty of Science, University of Colima, Bernal Díaz del Castillo 340, Colima, Colima 28045, Mexico; E-Mail: reyesgj@ucol.mx; 4 Faculty of Chemical Sciences, University of Colima, Km 9 Carretera Colima-Coquimatlán, Coquimatlán 28400, Colima, Mexico; E-Mails: lorenzo.gildo@gmail.com (L.G.-O.); hguillenbonilla@gmail.com (H.G.-B.)

**Keywords:** sensing properties, CoSb_2_O_6_, trirutile, chemical synthesis

## Abstract

Experimental work on the synthesis of the CoSb_2_O_6_ oxide and its CO_2_ sensing properties is presented here. The oxide was synthesized by a microwave-assisted colloidal method in presence of ethylenediamine after calcination at 600 °C. This CoSb_2_O_6_ oxide crystallized in a tetragonal structure with cell parameters *a* = 4.6495 and c = 9.2763 Å, and space group *P*4_2_/*mnm*. To prove its physical, chemical and sensing properties, the oxide was subjected to a series of tests: Raman spectroscopy, Scanning Electron Microscopy (SEM) and impedance (*Z*) measurements. Microstructures, like columns, bars and hollow hemispheres, were observed. For the CO_2_ sensing test, a thick film of CoSb_2_O_6_ was used, measuring the impedance variations on the presence of air/CO_2_ flows (0.100 sccm/0.100 sccm) using AC (alternating current) signals in the frequency-range 0.1–100 kHz and low relative temperatures (250 and 300 °C). The CO_2_ sensing results were quite good.

## 1. Introduction

Due to environmental concerns, the constant monitoring of the gases emitted to the atmosphere is highly important. Therefore, intense research on the gas-sensors area is carried out nowadays, especially regarding sensors based on semiconductor materials, like the oxides SnO_2_, ZnO, TiO_2_, WO_3_, LaFeO_3_ and CoAl_2_O_4_ [1–7]. Such materials show interesting sensing properties, remaining at the same time chemically stable in the presence of polluting gases. In recent years, oxides with a trirutile-type structure, like CoSb_2_O_6_ [8,9] and ZnSb_2_O_6_ [10,11], are being studied for gas-sensing applications with very good results. These results are mainly attributed to the nano-sized structure of the trirutile oxides, which therefore determines the best synthesis process (route) to produce them [9]. The ceramic method for the synthesis of oxides has been extensively used; however, this conventional process involves some disadvantages, for instance, it is necessary to heat the sample at elevated temperatures for a long time. Such treatment results in inhomogeneous materials, no control of the particle size and a decrease of the surface area. In order to overcome these problems, wet chemical routes can also be successfully employed, at relative low temperatures, yielding nanoparticles with unique microstructural features [12–14].

In addition, the response of a sensor in polluting gases atmospheres is strongly related to the chemical reactions occurring on the oxide surface; therefore, the microstructure determines the conversion efficiency of chemical interactions into electrical signals. So, it is very important to synthesize materials with optimal morphologies [1,15]. In this work we implemented a chemical synthesis route (the microwave-assisted colloidal method) to produce the CoSb_2_O_6_ oxide for gas sensing purposes. The employed synthesis route was found very convenient because a good stoichiometry control was possible, influencing positively on the microstructural characteristics of the oxide.

## 2. Experimental Section

### 2.1. Synthesis of Trirutile-Type CoSb_2_O_6_ Oxide

The trirutile-type structure CoSb_2_O_6_ oxide was synthesized based on the procedure described in [8,9]. However, in this work, these reagents were used: SbCl_3_ (Sigma-Aldrich), Co(NO_3_)_2_·6H_2_O (Mallinckrodt), 1 mL of ethylenediamine, and 5 mL of ethanol (Golden Bell). The resulting solution was evaporated by means of a microwave oven (JES769WK General Electric) at low power (180 W at 10–20 s intervals; the absorbed energy was calculated to be close to 85 kJ). The obtained precursor material was dried at 200 °C in static air for 8 h, then calcined in a muffle (Novatech) at 600 °C in static air for 6 h. In this process, a heating rate of 100 °C/h was employed.

### 2.2. Characterization of CoSb_2_O_6_ Powders

The calcined powders were analyzed by X-ray diffraction (XRD) at room temperature, using a D500 Siemens diffractometer (Siemens, Munich, Germany) with a Cu-Kα radiation (λ = 0.1518 nm). The 2θ scanning range was from 10° to 70° with a velocity of 1° min^−1^. The powders were also analyzed by Raman spectroscopy (Renishaw, Inc, Schaumburg, IL, USA) using a 1000B microRaman Renishaw system, calibrated with a silicon semiconductor with its characteristic Raman peak at 520 cm^−1^. The laser (excitation wavelength of 830 nm) was focused on the surface of the powders (spot size of approximately 20 μm) by means of a Leica optical microscope (DMLM; Leica microsystems, Deerfield, IL, USA) integrated to the microRaman system. The radiation energy on the sample was 4.5 mW 60 s. The morphology of the CoSb_2_O_6_ powders was analyzed by scanning electron microscopy (JEOL JSM-6390LV; Jeol, Inc., Dearborn, MI, USA).

The electrical characterization was made through dynamic tests of the impedance variation using a thick film made of oxide powders, which were set inside of a controlled tube-type furnace at temperatures of 250 and 300 °C. The impedance measurements were made by the two-tips technique using a computer controlled (with LabView 8.6, National Instruments) (Agilent 4263B device; Agilent Technologies, Inc, Santa Clara, CA, USA). The flow of the synthetic air/CO_2_ gases was controlled by a MKS 647C controller (MKS Instruments, Inc, Andover, MA, USA).

## 3. Results and Discussion

### 3.1. X-Ray Diffraction Analysis

**Figure 1.**
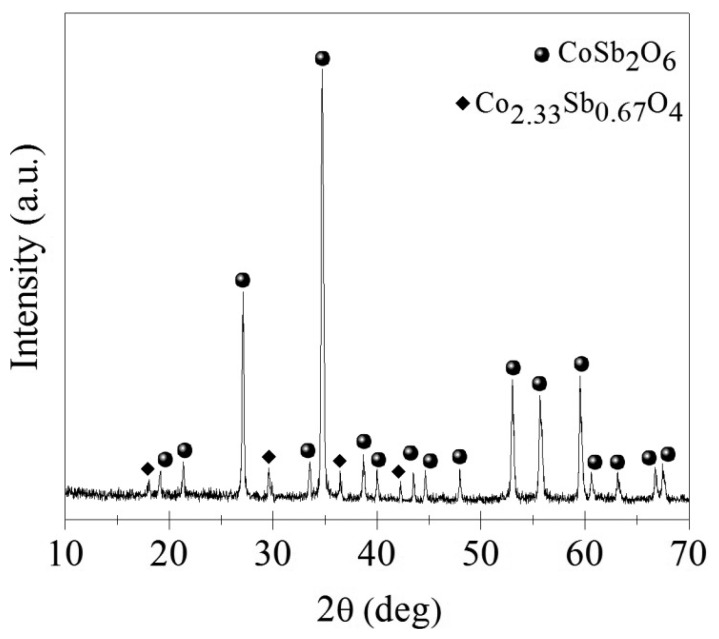
X-ray diffraction pattern of CoSb_2_O_6_ powders calcined at 600 °C in air.

Figure 1 shows an X-ray diffraction pattern of the powders prepared by the above described method.

The diffractogram shows the presence of the main phase corresponding to CoSb_2_O_6_, which was identified using the file ICDD 203094. According to this card, the CoSb_2_O_6_ oxide crystallized in a tetragonal structure with cell parameters *a* = 4.6495 and *c* = 9.2763 Å, and space group *P*4_2_/*mnm* (136) [16], which indicates that the oxide belongs to the family of trirutile-type structures [17]. In addition, the width of the peaks in the diffraction spectra is an indication of crystals of nanometric size; the low noise level indicates that the sample possesses a high crystallinity [18]. The estimated crystal size according to the Scherrer equation [19] for the CoSb_2_O_6_ powders was around 41 nm. Furthermore, low intensity peaks identified through the ICDD 87234 file, reveal a secondary phase: Co_2.33_Sb_0.67_O_4_ [9], localized on the angular positions 2θ = 17.8°, 2θ = 29.5°, 2θ = 36.2°, and 2θ = 42°.

Several other synthesis methods have been used for the preparation of oxides with trirutile-type structure. Larcher *et al.* [20] synthesized trirutile-type ASb2O6 materials (A = Ni, Cu, Co), following the solid-state reaction method, obtaining the structures at 800 °C. Michel *et al.* [21] obtained the CoSb2O6 oxide employing the solution-polymerization method at 700 °C. From the present work, and comparing with other studies, it can be stated that the obtention of the CoSb_2_O_6_ oxide is very feasible at temperatures significantly lower than those used by the authors above mentioned, by means of the synthesis route proposed here (the microwave-assisted colloidal method).

### 3.2. Raman Spectroscopy Analysis

**Figure 2.**
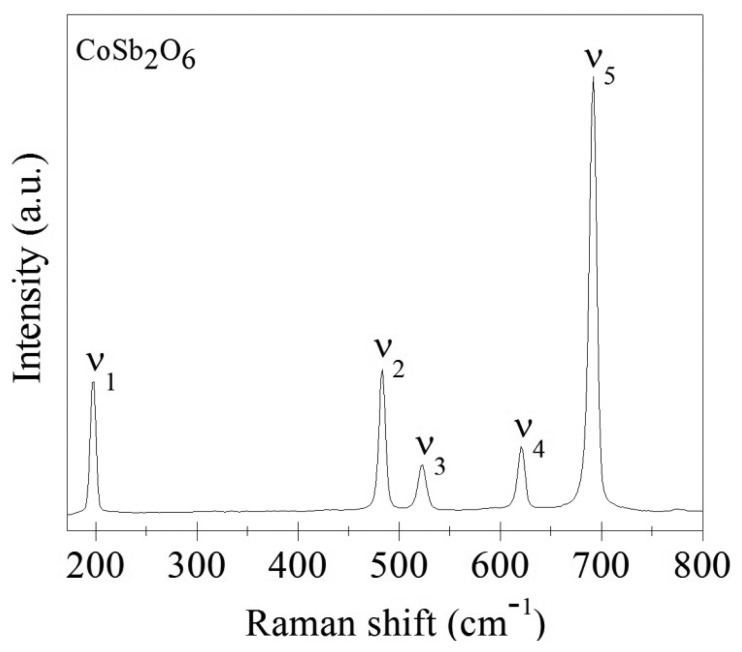
Raman spectrum of CoSb_2_O_6_ powders calcined at 600 °C in air.

The Raman analysis (Figure 2 and Table 1) shows the vibrations modes A_1g_, B_1g_, B_2g_ and E_g_ at the range 190–800 cm^−1^. Band *v*_1_ has a mean intensity ∼194 ± 3 cm^−1^ and is due to the vibrations of the crystalline network with A_1g_ symmetry. Band *v*_2_, at ∼478 ± 3 cm^−1^, is due to the deformation vibration of the Co-O bond, also with A_1g_ symmetry. Band *v*_3_, at ∼518 ± 3 cm^−1^, can be attributed to the combination of the stretching and coupling vibrations of the Sb-O bonds, with symmetry E_g_. Band *v*_4_, at ∼617 ± 3 cm^−1^, is due to the asymmetric stretching vibration of the Sb-O bond, with symmetry E_g_. Band *v*_5_, at ∼641 ± 3 cm^−1^, showing a high intensity, is due to the symmetric stretching vibration of the Sb-O bond, with E_g_ symmetry. These results are in agreement with [22] and [23], and support the correct characterization of the oxide.

### 3.3. Scanning Electron Microscopy Analysis

**Figure 3.**
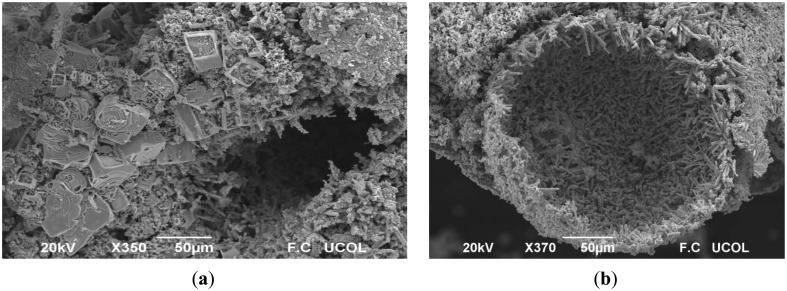

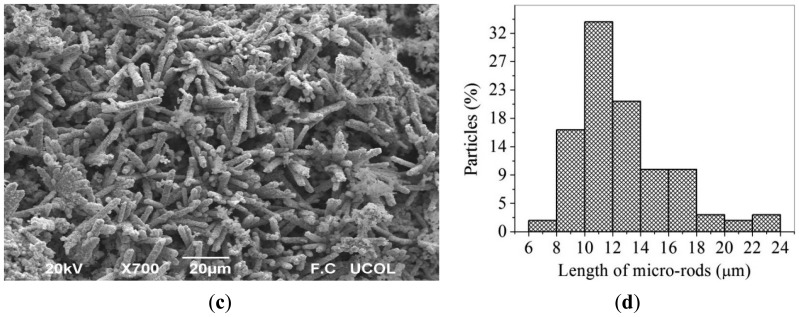
SEM (scanning electron microscopy) images of CoSb_2_O_6_ powders at different magnifications: (**a**) 350X; (**b**) 370X; and (**c**) 700X; (**d**) length distribution of the micro-rods.

Figure 3 shows photomicrographs of the CoSb_2_O_6_ oxide at several magnifications: (a) 350X, (b) 370X, and (c) 700X. The discerned particle shapes are rectangular micro-bars with sizes: base ∼28.5 μm, length ∼42 μm and height ∼13 μm. Around these bars, some smaller structures in form of micro-rods, growing in different directions, are also discernible. The micro-rods growth, with an estimated diameter of ∼2.7 μm, is attributed to the increasing of the temperature and the effects caused by the ethylenediamine. These micro-rods tend to aggregate to form a hollow hemisphere (see image b). The granulate surface of the micro-rods is probably due to the released gases from organic material during the thermal decomposition in the oxide's synthesis [24]. The size distribution of the micro-rods (also shown in Figure 3d) depicts a length range of 6–24 μm with a standard deviation of 3.4 μm and an average length of ∼12.7 μm. The role of ethylenediamine in the formation of 1D structures of II–VI semiconductors, like nanorods and nanowires, has been discussed in previous works [25,26]. The ethylenediamine is incorporated first into the inorganic framework and then escapes from it to form particles of desired morphologies [26]. In this work, we found that the presence of ethylenediamine in the CoSb_2_O_6_ synthesis leads to the growth of micro-rods. In fact, we reported in a previous work [27] the obtention of trirutile-type MgSb_2_O_6_ with different morphologies, micro-rods and micro-plates among them, by the use of ethylenediamine.

On the other hand, the preparation of different inorganic compounds by means of colloidal routes has been widely studied by Matijevic [28]. Some authors report the obtention of morphologies similar to those here reported, which originate from the growth process of stable nuclei of the colloidal systems [28,29]. These morphologies are in agreement with the crystallization principles proposed by LaMer and Dinegar [9,24,27,30].

### 3.4. Impedance Measurements and Gas Sensing Properties

**Figure 4.**
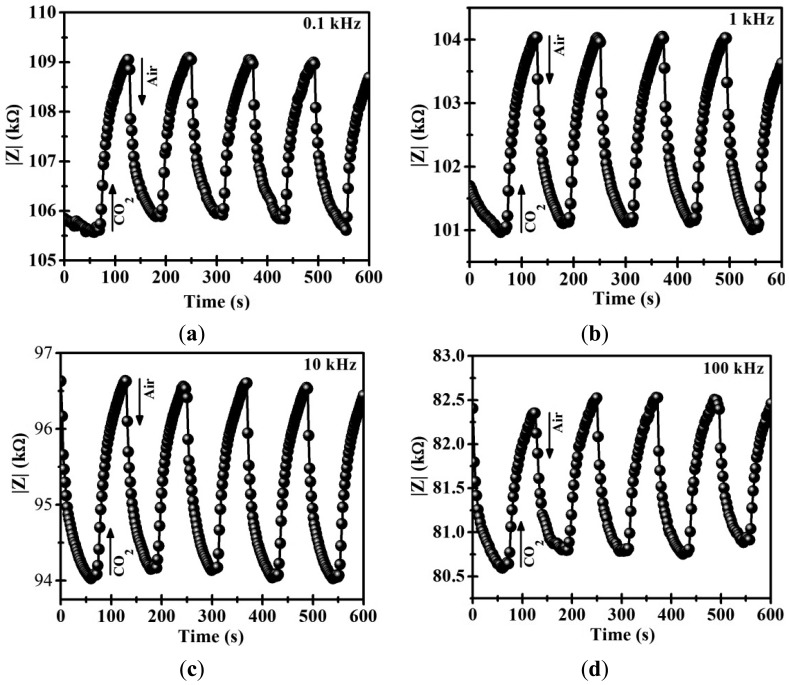
Dynamic response of the CoSb_2_O_6_ oxide subjected to CO_2_ flows at 250 °C and different frequencies: (**a**) 0.1 kHz; (**b**) 1 kHz; (**c**) 10 kHz; and (**d**) 100 kHz.

To test the gas sensing properties of the CoSb_2_O_6_ oxide, a thick film (thickness ∼500 μm) was employed for measuring its impedance variation in presence of air/CO_2_ flows (0.100 sccm/0.100 sccm). Experiments at four frequencies (AC (alternating current) signals) were conducted in this study: 0.1, 1, 10 and 100 kHz, and at two different temperatures, namely, 250 and 300 °C. The process to perform the dynamic response experiments consisted of three stages: (1) for each frequency and temperature, synthetic air was flowed over the film's surface during approximately 5 min in order to stabilize it; (2) a CO_2_ flow was applied on the material, recording the impedance (|*Z*|) variations; (3) after one minute, synthetic air was flowed again over the thick film, returning the impedance |*Z*| to the initial state. Figure 4 shows the dynamic tests results of the CoSb_2_O_6_ oxide at 250 °C, and Table 2 shows the calculated variations. In all four tests (0.1, 1, 10 and 100 kHz), the magnitude of the impedance (|*Z*|) changes in an alternating manner when the gas flow changes from air to CO_2_. These results reveal that the material exhibits sensitivity and repeatability in CO_2_ detection. In particular, at the 0.1 kHz frequency, the change of the magnitude |*Z*| was ∼3.23 kΩ on average (Figure 4a); while at the 1 kHz frequency, the recorded change was ∼2.91 kΩ on average (Figure 4b). On the other hand, at the 10 kHz frequency, the magnitude of the impedance changes ∼2.43 kΩ on average (Figure 4c); however, using a high frequency of 100 kHz, the magnitude of the impedance is reduced to an average value of ∼1.71 kΩ (Figure 4d).

In addition, Figure 4 shows the fast response and recovery times recorded during the exposure to the CO_2_ gas at 250 °C. The response times, measured at 90% of the full response, were 22.38, 21.56, 20.32 and 18.19 s at the frequencies 0.1, 1, 10 and 100 kHz, respectively, while the recorded recovery times were 21.36, 20.06, 19.06 and 17.95 s at the same frequencies, respectively (See Table 2). From these results, CoSb_2_O_6_ thick films respond faster and with a shorter recovery time at the higher frequency (100 kHz); however, upon increasing the frequency, smaller |*Z*| values were registered.

**Figure 5.**
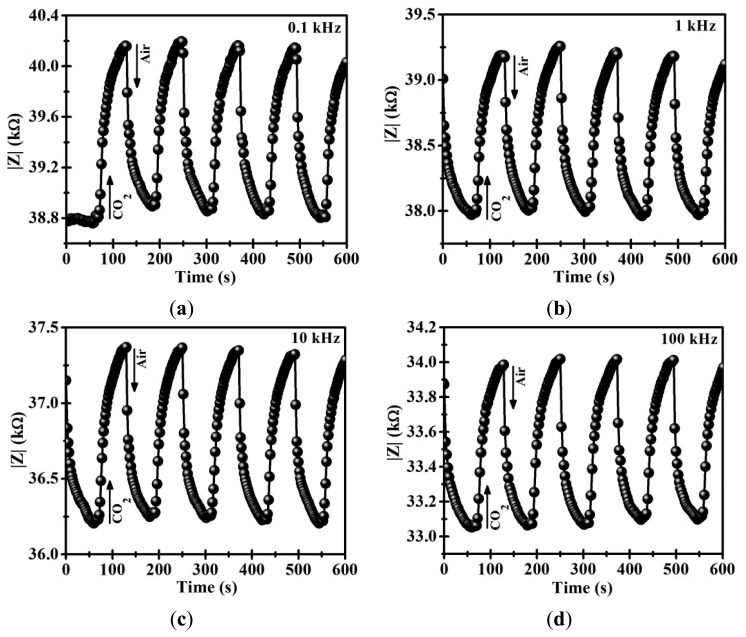
Dynamic response of the CoSb_2_O_6_ oxide subjected to CO_2_ flows at 300 °C and different frequencies: (**a**) 0.1 kHz; (**b**) 1 kHz; (**c**) 10 kHz; and (**d**) 100 kHz.

Figure 5 shows the results of the dynamic tests on the CoSb_2_O_6_ oxide at 300 °C with the same frequencies and gases flows, and the calculated variations are shown in Table 3. Similar tendencies to those shown in Figure 4 were recorded. However, at 300 °C, the changes of the magnitude |*Z*| were ∼1.29, ∼1.18, ∼1.09 and ∼0.921 kΩ, at the frequencies 0.1, 1, 10 and 100 kHz, respectively. In addition, the response and recovery times were reduced to values less than 20 s due to the effect of the temperature. In this case, the response times were 17.32, 16.30, 15.00 and 14.18 s, while the recovery times were 16.18, 15.00, 13.93 and 13.00 s at the same frequencies, respectively (see Table 3). Clearly, at higher temperatures and frequencies applied, the CoSb_2_O_6_ sensor exhibits faster response and shorter recovery times.

**Figure 6.**
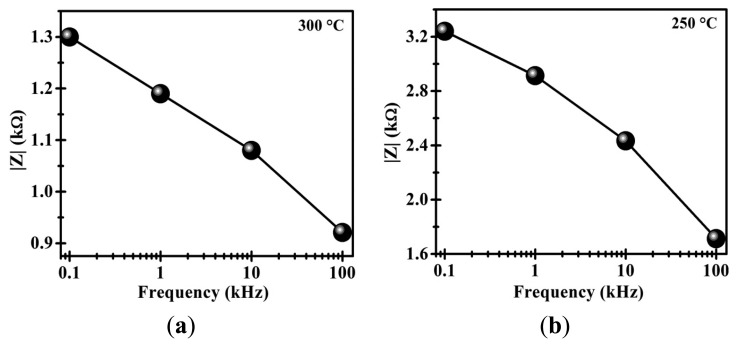
Impedance |Z| *vs.* frequency tests of the CoSb_2_O_6_ oxide at (**a**) 250 °C and (**b**) 300 °C with air/CO_2_ flows.

**Figure 7.**
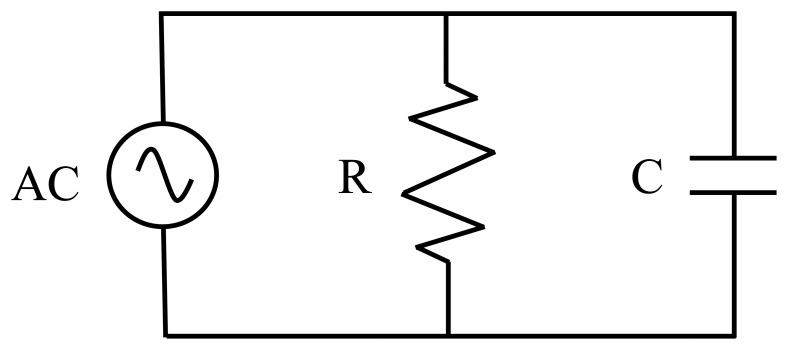
Equivalent RC parallel circuit for the CoSb_2_O_6_ sensor.

To discern the effects of frequency and temperature on the CoSb_2_O_6_ ability to detect air/CO_2_, Figure 6 shows graphically the above mentioned variations. Clearly, increasing the frequency and the temperature, the impedance drops considerably, which is a characteristic of semiconductor materials. At 250 °C, the average |*Z*| is 2.52 kΩ and at 300 °C, it is 1.12 kΩ. The oxide therefore behaves like a low-pass filter and can be modeled as a parallel RC circuit (see Figure 7). This circuit is made up of the alternating current (AC) source, a resistance (R) and a capacitor (C) [8]. In addition, the behavior of the dynamic response obtained at 250 and 300 °C can be reproducible. This suggests that this material can reliably be applied as a gas sensor under air/CO_2_ flows [31].

Comparing the results of Figures 4 and 5 with those reported in previous works corresponding to the CoSb_2_O_6_ oxide, a better performance as gas sensor was obtained in the present work. For example, nanostructured CoSb_2_O_6_ microspheres have been synthesized employing a non-aqueous method in presence of n-dodecylamine [9]. These CoSb_2_O_6_ microspheres were sensitive to detect 400 ppm of CO_2_ at 400 °C with a response time of 130 s and a recovery time of 22 s. Also, the variation in electrical resistance recorded was 130 Ω. In addition, the dynamic response of CoSb_2_O_6_ prepared by solution-polymerization method has been reported [21]. In this paper, a CO_2_ flow (100 cm^3^/min) produces an increase in electrical resistance of 900 Ω in 3 min (410 °C). However, the CoSb_2_O_6_ oxide synthesized in this work exhibits good sensing properties at lower temperatures (250 and 300 °C) to those reported in the previous works. Additionally, a faster response, shorter recovery times, better repeatability and larger impedance changes were obtained. This gas response can be attributed to the microstructure found in this work, and its nanocrystalline nature.

Moreover, from Figures 4 and 5 the rise of |*Z*| during the flow of CO_2_ indicates a p-type semiconductor [31,32]. In general, the gas-sensing mechanism in semiconductor materials is based on the electric resistance change (conductance) produced by the electron transfer occurring during the chemical adsorption [32]. In the presence of a gas, such as the one used in this study (CO_2_), electrons are provided to the oxide surface and they combine with holes. Therefore, the concentration of electrical charge carriers (holes) is reduced and the magnitude |*Z*| increases. The process of CO_2_ adsorption on metal oxide surfaces can involve the formation of several carbon species, such as bicarbonate and carbonates [33]. These carbon species are removed when air is injected. Then, the magnitude |*Z*| reached its original value.

Depending on the type of the semiconductor, the concentration of the charge carriers on the surface can be increased or decreased [34]. The thickness of the charged *L_S_* outer layer can be defined as [34]:

(1) $$L_{S}=L_{D}\sqrt{\frac{eV_{S}^{2}}{kT}}$$

where *L_D_* is the Debye length, *e* is the charge of the electron, *V_S_* is the potential of the surface, *k* is the Boltzmann constant, and *T* is the temperature. Usually, the *L_S_* values are between 1 and 100 nm [34].

According to some authors, the charged outer layer mainly depends on the gas pressure and the gas concentrations. These results of the changing resistance value and the conductivity variation of the semiconductor can be consulted in [35]. Due to this, the conductivity strongly depends on the size of the crystal (*D*), thus giving rise to three possible scenarios [36]:

(1) If *D* ≫ 2*L_S_*, the conductivity is limited by the Schottky barrier at the particle border; thus, the gas detection does not depend on the crystal size (*D*),
(2) If *D* = 2*L_S_*, the conductivity and the gas sensing depend on necks formed by the crystals, and
(3) If *D* < 2*L_S_*, the conductivity depends on the crystal size [36,37]. Based on these three scenarios, it can be said that the smaller the size of the particle during its synthesis, the greater the surface area obtained and, therefore, a better gas adsorption [34].

In addition, the good electric response of the CoSb_2_O_6_ under a controlled air/CO_2_ flow can be to a great extent explained by the presence of microstructures obtained during the process of synthesis.

## 4. Conclusions

Desirable trirutile-type CoSb_2_O_6_ structures for gas-sensing applications, like micro-rods and micro-bars, were successfully prepared by a wet-chemical synthesis route at relative low temperatures. The results of this work, regarding the sensing properties of the synthesized material, show that at different frequencies, the dynamic response improves under air/CO_2_ flows. The CoSb_2_O_6_ thick films respond faster and with a shorter recovery time at higher temperatures and frequencies (100 kHz and 300 °C). Also, the results indicated that increasing the frequency, smaller |*Z*| values were obtained. The uniformity of the dynamic tests reflects the good impedance response during the adsorption of the CO_2_. According to this, the CoSb_2_O_6_ oxide is a strong candidate to be applied as an environmental gas sensor, especially when synthesized by our proposed method.

## Figures and Tables

**Table 1.** Vibration modes of the CoSb_2_O_6_ oxide.

**Mode**	**Raman Displacement (cm^−1^)**	**Symmetry**	**Vibration Type**	**Molecular Bond**
***v*_1_**	∼194 ± 3	A_1g_	Crystalline network	
***v*_2_**	∼478 ± 3	A_1g_	Deformation	Co-O
***v*_3_**	∼518 ± 3	E_g_	Stretching and Coupling	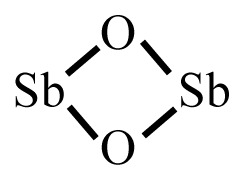
***v*_4_**	∼617 ± 3	E_g_	Stretching (asymmetric)	Sb-O
***v*_5_**	∼641 ± 3	E_g_	Stretching (symmetric)	Sb-O

**Table 2.** Variations of the impedance measurement at 250 °C.

**Frequency (kHz)**	**Mean Impedance (kΩ)**	**Response Time (s)**	**Recovering Time (s)**
0.1	3.23	22.38	21.36
1	2.91	21.56	20.06
10	2.43	20.32	19.06
100	1.71	18.19	17.95

**Table 3.** Variations of the impedance measurement at 300 °C.

**Frequency (kHz)**	**Mean Impedance (kΩ)**	**Response Time (s)**	**Recovering Time (s)**
0.1	1.29	17.32	16.18
1	1.18	16.30	15.00
10	1.09	15.00	13.93
100	0.921	14.18	13.00
